# Public involvement in health research systems: a governance framework

**DOI:** 10.1186/s12961-018-0352-7

**Published:** 2018-08-06

**Authors:** Fiona Alice Miller, Sarah J. Patton, Mark Dobrow, Whitney Berta

**Affiliations:** Institute of Health Policy, Management and Evaluation, University of Toronto, 155 College Street, 4th Floor, Toronto, ON M5T 3M6 Canada

**Keywords:** Public involvement, Community-based research, Health research systems, Research governance, Research policy

## Abstract

**Background:**

Growing interest in public involvement in health research has led to organisational and policy change. Additionally, an emerging body of policy-oriented scholarship has begun to identify the organisational and network arrangements that shape public involvement activity. Such developments suggest the need to clearly conceptualise and characterise public involvement in health research in terms of governance.

**Methods:**

We drew on an established health research system framework to analyse governance functions related to public involvement, adapting scoping review methods to identify evidence from a corpus of journal papers and policy reports. We drew on the logics of aggregation and top down configuration, using a qualitative interpretive approach to combine and link findings from different studies into framework categories.

**Results:**

We identified a total of 32 scholarly papers and 13 policy reports (*n* = 45 included papers) with relevance to governance for public involvement. Included papers were broadly consonant in identifying the need for activity to specify and support public involvement across all four governance functions of stewardship, financing, creating and sustaining resources, and research production and use. However, different visions for public involvement, and the activity required to implement it and achieve impact, were particularly evident with respect to the stewardship function, which seeks to set overall directions for research while addressing the potentially competing demands of a system’s many constituents.

**Conclusions:**

A governance perspective has considerable value for public involvement in health research systems, supporting efforts to coordinate and institutionalise the burgeoning public involvement enterprise. Furthermore, it highlights challenges for what is, ultimately, a highly political intervention, suggesting that diverse publics must be both involved within health research systems and enrolled as governors of them.

**Electronic supplementary material:**

The online version of this article (10.1186/s12961-018-0352-7) contains supplementary material, which is available to authorized users.

## Background

Public involvement in health research is increasingly seen as essential to the legitimacy, relevance and quality of the research enterprise, enabling research to better account for the needs of service users and caregivers, to respond to the demands of the lay communities affected by research practices and results, and to respect the imperatives of democratic accountability in service of the public interest [1–4]. Accordingly, there has been a marked growth of public involvement activity, with increased reliance on representatives of diverse publics as advisors to, or investigators within, individual research projects [3–6], and growing interest in involving public members in advising on and setting priorities for research funding [7]. This has been accompanied by an explosive growth of scholarship reporting on the rationales, methods and impacts of public involvement activity [8–12].

While much of this effort has been motivated by individual researchers and members of the public, it has also been conditioned by policy effort. Public sector authorities in many countries, including funding agencies and oversight bodies, are increasingly seeking to encourage, coordinate or evaluate such initiatives [13, 14]. Organisations with the mandate to facilitate public engagement have been established and public involvement policies increasingly inform the efforts of research producers, including individual researchers as well as producer organisations such as universities, hospitals or research institutes [13–18]. Such policy developments have also attracted scholarly interest, and an emerging body of policy-oriented work has begun to explore organisational and jurisdictional efforts related to public involvement as well as the arrangements and institutions that direct the health research enterprise and condition the potential for the successful implementation of the public involvement agenda [2, 19].

Policy efforts to direct public involvement in health research, alongside an emerging body of policy-oriented scholarship, suggest the need to clearly conceptualise and characterise public involvement in health research in terms of governance; that is, the way rules, norms and actions are structured, sustained and regulated to condition the operation and impact of public involvement activity. The term ‘governance’ differs from ‘government’ in highlighting the distributed nature of authority and the many ways that individuals and groups “*organize themselves to achieve agreed goals*” [20]. Governments are looked to as essential actors from this perspective, but so too are authorities in the para-public or private sectors, creating opportunities for “*good governance*” [21] alongside challenges for democratic and formal accountability [22].

Governance efforts in the health sector are increasingly informed by ‘systems thinking’. For example, WHO has issued guidance on Systems Thinking for Health System Strengthening [23] as well as the WHO framework for conceptualising ‘health research systems’ (HRS) to ensure “*knowledge for better health*”, which has particular salience for research policy [24]. Emerging from the international Commission on Health Research for Development, the HRS literature aims to support countries, and the global community, to build and sustain systems of research “*involving people, institutions and processes*” that serve health systems and support population health and health equity [25]. The WHO World Health Report of 2013 reinforced the value of the ‘systems’ perspective on health research, noting that, “*To make the best use of limited resources, systems are needed to develop national research agendas, to raise funds, to strengthen research capacity, and to make appropriate and effective use of research findings*” [26].

The HRS framework is highly relevant to governance for public involvement. The framework was designed in recognition of the multiplicity of arrangements, expectations, obligations and incentives in research contexts that are often “*fragmented, competitive,* [and] *highly specialized*” [27], which is salient to public involvement, given the challenges it may pose to dominant interests and usual practice [2]. In a related vein, the framework is attentive to the diversity of stakeholders within a HRS, and the navigation of stakeholders’ multiple, and not always compatible, interests [28]. Further, the HRS framework expresses an openness to public involvement, including where communities lead their own research, as with emancipatory research designs [25, 28]. Finally, the HRS framework is clear regarding the imperative to attend to health research beyond healthcare in order to address the social determinants of health and the demands of health equity [25, 27].

Thus, to inform efforts to foster public involvement across the provincial health research enterprise in Ontario, Canada, we sought to explore the relevance of the HRS framework to governance for public involvement. We drew specifically on the conceptual framework developed by Pang et al. [27] to consider the principal functions and associated operational components of a HRS. The framework provided the basic tools for conceptualising governance and for operationalising our review of relevant evidence.

## Methods

We drew on scoping review methodology to identify a corpus of scholarly papers and policy reports with relevance to governance for public involvement within HRS [29, 30]. We included conceptual and empirical papers and did not assess quality or exclude papers on that basis. Given time and resource constraints, we iteratively reviewed only one database, and supplemented the database search with a targeted environmental scan of policy reports relevant to public involvement from major public sector research organisations that fund health research or support public involvement in health research across selected jurisdictions. Finally, the project team was advised in the review and targeted scan by a committee of local experts from the health sector, who participated in team meetings and advised on project execution [31].

### Database search and selection strategy

We began with a series of targeted searches to understand the state of the literature and refine our research questions. Then, in consultation with a research librarian, we developed a strategy for searching electronic databases, translating search concepts into keywords and medical subject heading (MeSH) terms using common indexing practices. From December 2015 through February 2016, we searched the biomedical electronic database, Ovid MEDLINE, from its inception through December 2015. Search terms were compiled and tested several times to capture potentially relevant articles. Due to time and resource constraints, no additional database searches were conducted. However, given the rapidly evolving nature of the field, we updated our search in 2017, using the same search terms in the same database, limited to articles published between December 2015 and June 2017. We report here on these combined searches to June 2017, inclusive (Additional file 1: Search terms).

Two reviewers independently screened the titles and abstracts for potential inclusion, retaining all papers that were included by either reviewer. The same two reviewers then independently assessed the full text of potentially relevant papers for eligibility for final inclusion. Final decisions on inclusion were made through discussion with a third reviewer.

We included all original studies of any design, including all non-empirical papers (commentaries, editorials) published in the English language that discussed how to organise, systematise or oversee the involvement of patients, members of the lay public or communities in health research within organisations or jurisdictions. Specifically, we included papers that were (1) concerned with ‘publics’ – patients (clients, consumers, informal caregivers organised groups), members of the lay public, lay communities; (2) about ‘engagement’/‘involvement’ of publics, including community-based participatory action and related research; (3) about health research of any sort (basic, health services, health systems, etc.); and (4) relevant to health research governance and ‘systems’, by addressing considerations or efforts at organisational or inter-organisational (i.e. meso) or jurisdictional (i.e. macro) levels. Articles specific to involvement in healthcare systems or health service delivery were excluded, as were articles concerned with public involvement in science that lacked a clear focus on health research. To identify additional potential papers, we reviewed reference lists from included articles and conducted further searches using the Google Scholar ‘cited by’ and the MEDLINE PubMed ‘related articles’ features. The Preferred Reporting Items for Systematic Reviews and Meta-Analyses (PRISMA) criteria were used to help guide the conduct and reporting of the review [32].

### Targeted environmental scan of policy reports

Informed by findings from the MEDLINE database search and suggestions by Advisory Committee members, we identified major organisations with leadership roles with respect to funding health research or supporting public (patient, community, lay public) involvement in health research in selected jurisdictions (United Kingdom, United States, Australia and Canada), and searched online using Google for relevant policy reports (Additional file 2: list of reviewed organisations). Only key framework, guidance or evaluative documents were included.

### Data extraction, analysis and synthesis

We aimed to use the HRS framework developed by Pang et al. [27] as a data extraction template for the corpus of collected papers, seeking evidence across the framework’s four domains and nine embedded operational components. Given the utility of the existing framework for an international community of practice, we aimed to make few modifications. However, some adaptations to ensure that the framework was relevant to public involvement were necessary. Adaptations were based mainly on an a priori logic but also arose from reflection on the data captured in each category.

We drew on the ‘logics’ for mixed methods-mixed research synthesis identified by Sandelowski et al. [33] to analyse the dataset. Specifically, we adopted the logic of research synthesis by aggregation, which entails the assimilation of findings considered to address the same relationship or connection between two or more aspects of a target phenomenon. Additionally, we adopted the logic of research synthesis by configuration, which involves a more theoretical rendering of the data reviewed, arranging diverse findings to forge a new interpretation. Our approach to configuration was ‘top down’, as we sought to interpret studies in light of a prior conceptual framework [33]. We analysed the data thematically, using a qualitative interpretive approach and drawing on the traditions of constructivist grounded theory to merge and link findings from different studies into common categories [34–36].

## Results

### Overview of included papers

**Fig. 1:**
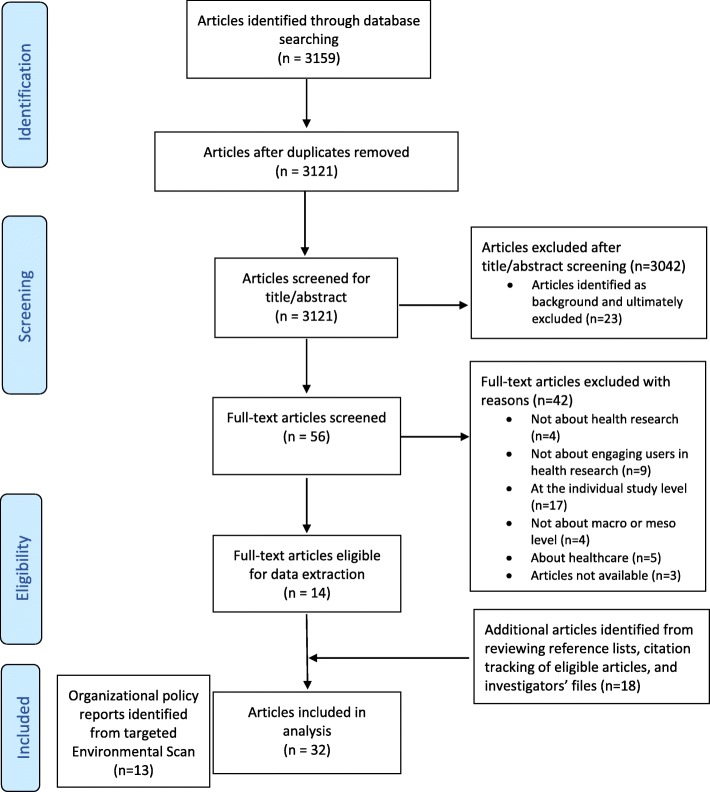
PRISMA flow diagram

The database search identified 3121 unique papers, of which 56 papers were retained after title and abstract screening. Of these, 14 were retained after full text screening. A further 18 papers were added after reviewing reference lists of included papers and conducting supplementary citation searches and from investigators’ files (Additional file 3: full list of 32 scholarly papers). The targeted environmental scan identified 13 policy reports, for a total of 45 included papers (Table 1; Additional file 4: list of 13 policy reports; Fig. 1 for PRISMA diagram).

**Table 1:** Included papers (*n* = 45)

	Scholarly papers	Policy reports^a^
Country focus	∙ *n* = 14, United Kingdom	∙ *n* = 4, United Kingdom
	∙ *n* = 7, United States	∙ *n* = 4, United States
	∙ *n* = 3, Australia	∙ *n* = 3, Canada
	∙ *n* = 4, European Union	∙ *n* = 2, Australia
	∙ *n* = 3, Global	
	∙ *n* = 1, Canada	
Research design	∙ *n* = 14, Empirical (surveys, qualitative interviews)	∙ Not available
	∙ *n* = 18, Commentary (opinion piece or description)	
Organisational focus or source	∙ *n* = 12 About funding agencies or programmes such as national funding bodies, major medical charities or specific funding programmes	∙ *n* = 10, National funding bodies that support a wide range of health and medical research (discovery, epidemiology, population health, clinical, health services research)
	∙ *n* = 3 About academic organisations such as universities or research hospitals	∙ *n* = 3, Organisations that support public engagement for a wide range of health research
	∙ *n* = 6 About organisations that represent or support the public, some of which also commission or raise funds for research or produce research	
	∙ *n* = 11 About multiple research organisations, networks or systems	
Publication year	∙ *n* = 15, 2000–2009	∙ *n* = 1, 2000–2009
	∙ *n* = 17, 2010–2017	∙ *n* = 12, 2010–2017

^a^Counts are derived from a targeted search; as such, the proportions should not be taken to indicate the volume of work on this topic by country

Most included papers reported on, recommended or described the engagement efforts of various research organisations such as funding agencies, academic organisations or organisations that represent publics. Only a small proportion explicitly aimed to explore public engagement as a policy effort or within health research networks, structures or systems that spanned organisations [2, 19, 37], and only one referenced a HRS conceptual framework [38]. Commentary was more common than empirical research.

### Governing public involvement for HRS

We identified activities relevant to public involvement across all functions in the HRS framework. Further, we identified roles related to public involvement for both governments as well as research organisations that act as public or para-public stewards such as research funding agencies, research producer organisations (e.g. universities, hospitals) and organisations or organisational units that aim to support public involvement [27] (Table 2).

**Table 2:** Functions and operational components of health research systems (HRS) – original and adapted

Function	Original HRS Framework	Adapted HRS Framework
Stewardship	∙ Define and articulate vision for a national HRS	∙ Define and mobilise a vision for public involvement in a HRS
	∙ Identify appropriate health research priorities and coordinate adherence to them	∙ Define the role of public involvement in identifying appropriate health research priorities and coordinating adherence to them
	∙ Set and monitor ethical standards for health research and research partnerships	∙ Set and monitor ethical standards for health research
	∙ Monitor and evaluate the HRS	∙ Monitor and evaluate public involvement in the HRS
Financing	∙ Secure research funds and allocate them accountably	∙ Secure research funds to support public involvement
		∙ Define the role of public involvement in accountably allocating research funds
Creating and sustaining resources	∙ Build, strengthen and sustain the human and physical capacity to conduct, absorb and utilise health research	∙ Define the human resource requirements to build, strengthen and sustain public involvement capacity
		∙ Define the organisational requirements to build, strengthen and sustain public involvement capacity
Producing and using research	∙ Produce scientifically valid research outputs	∙ Define the role of public involvement in producing and using research
	∙ Translate and communicate research to inform health policy, strategies, practices and public opinion	
	∙ Promote the use of research to develop new tools (drugs, vaccines, devices and other applications) to improve health	

Adapted from Pang et al. [27].

#### Stewardship

According to Pang et al. [27], the first core function of an effective HRS is stewardship.

##### Define and mobilise a vision for public involvement

As anticipated by the Pang et al. framework [27], ‘vision’ was identified as important for advancing public involvement in health research, including at the level of the jurisdiction or research organisation. Yet, the substance of such visions and their implications varied (Table 3). On the one hand, there was a general endorsement of efforts to formalise and implement clear visions, which was particularly emphasised in policy reports [15–17], though some scholarly papers also endorsed the value of well-defined visions that were supportive of public involvement.

**Table 3:** Organisational and policy visions for public involvement – role, politics and nature

Role of vision	∙ Organisational vision to govern own involvement or issue guidance to develop value-based involvement through organisational policies and strategies [15–17, 56, 63]
	∙ Value of clear visions to support public involvement at national level and in organisations [52, 53, 62]
Politics of vision	∙ Potential for visions to advance organisational self-interest and sustain status quo [19]
	∙ Rise of public involvement reflecting increased public scepticism or concern [39, 40]
	∙ Rise of public involvement reflecting particular political imperatives that advantage some publics and disadvantage others (e.g. New Labour, patient choice, business development) [19, 40–42]
Nature of vision	*Key constituents:*
	∙ Patients as service users and persons affected by illness (also caregivers and families) with experience-based knowledge of health conditions, treatments and care pathways – in policy reports and scholarly papers [1, 4, 15, 16, 43]
	◦ Interests in diverse types of health research [2, 53, 62]
	∙ Communities with collective expertise derived from history or identity, often involving social disadvantage, including inequities in access to care and disparities in social opportunity or health outcomes – in scholarly papers [38, 41, 44–46]
	◦ Specific interests in population health, health equity and social determinants of health [45, 51, 61]
	*Approach to involvement:*
	∙ Partnership and shared control [49, 50] or participatory and action-oriented research
	∙ Involvement spectrum, including communication about research, fundraising for research and participation in research [13, 17, 52]

Several scholarly papers reflected critically on the source and implications of organisational or policy visions for public involvement. At the national level, government attention to public involvement in health research could be seen as a response to public scepticism or concern [39, 40], but also as a resource for political platforms such as patient choice in healthcare or democratic accountability in general [19, 40]. Importantly, such political visions did not empower all publics equally [41, 42]. Additionally, some authors cautioned that visions for public involvement could be used to support the self-interest of research organisations more than organisational or system change [19].

Further, visions for public involvement differed substantively in the way publics and public involvement were conceived of. Visions differed, first, with respect to the type of publics to engage – whether patients or communities, with attention to communities more present in scholarly literature than policy reports. These different constituents offered various forms of expertise and implied different types of research. Patients were understood to provide expertise derived from personal experience with health conditions, such that patient involvement encouraged attention to the various types of health research that could inform improved health outcomes and high-quality care [1, 4, 15, 16, 43]. Community expertise was understood to emerge from collective conditions and opportunities relevant to population health, and scholarly papers drawing on traditions of community-based participatory action research encouraged attention to health inequities and the social determinants of health [38, 41, 42, 44–46]. In addition to these two core publics, lay persons were occasionally referenced as general or disinterested publics to involve [47]. Finally, other research stakeholders (e.g. health professionals) were sometimes included in discussions of public involvement [47, 48], without necessarily clarifying the distinctive significance of patient, community or lay public expertise (Additional file 5).

Visions also differed in terms of the types of involvement to pursue. While the scholarly literature generally anticipated high levels of public involvement [49, 50] and the literature on community involvement envisaged a specifically community-based, participatory and action-oriented approach [46, 51], policy reports were agnostic and typically referenced a spectrum of involvement levels, including activities such as communication about research, fundraising for research and participation in research [13, 17, 52].

Finally, some scholars considered the question of vision implementation and impact (Table 4). Several papers pointed to the importance of mechanisms to encourage visions to be implemented, including policy instruments at the national level that recommended, incentivised or obliged action [4, 37, 40, 48], as well as the policies or strategies of individual research producer organisations [4, 53–55]. Further, some scholars highlighted the limited degree of organisational or system change realised to date, identifying the partial ways in which publics have been embedded structurally with influence on outcomes, operationalised as partners in processes of ‘user led’ or ‘co-produced’ research, or able to successfully challenge dominant epistemological models of science that privilege professional scientific expertise [19, 37, 39]. These works also offered insight into the organisational actors and network processes that enable and condition public involvement, with key roles for ‘intermediary organisations’ [2], such as research funding bodies, research producing organisations and public involvement support organisations, in channelling and structuring public involvement activity [19].

**Table 4:** The implementation and impact of visions for public involvement

Implementation of vision	∙ Legislation or policy at national or supra-national level to encourage or require public involvement [4, 37, 40, 48]
	∙ Policies or strategies that mobilise public involvement within research organisations [4, 53–55]
	◦ Through consumer-led research organisations [68]
	◦ Through stewardship by research producers (universities, medical schools) [44, 61, 65]
Impact of vision	∙ The extent and nature of public involvement conditioned by organisational arrangements and network processes, including ‘intermediary organisations’ such as governmental research councils, private research financiers and social research institutes or departments working on the democratisation of science [2, 19]
	∙ Stabilised arrangements limit disruption of usual practice and emancipatory approaches with ‘professionalised’ publics and lack of representation and diversity [2, 37]

##### Defining the role of public involvement in identifying appropriate health research priorities and coordinating adherence to them

The included papers endorsed Pang et al.’s [27] view of priority-setting as a key stewardship function and highlighted the importance of public involvement in such processes (Table 5). For most, discussion of the issue centred on the need for public involvement in priority-setting and the expectation that involvement would make a difference with respect to the priorities selected [1, 7]. These discussions were typically focused at the level of the research organisation, with recommendations to increase public involvement in priority-setting [15, 16, 56], as well as descriptions of the ways different funding organisations had done so [6, 48, 57–59]. Less commonly, public involvement in priority-setting was discussed at the national level, and with respect to a specific moral purpose – that of health equity [60]. Discussions of approaches to involving publics in priority-setting were typically descriptive [57–59]. However, some authors cautioned about the need for more attention to the implications of different structures and practices of involvement for research priority-setting [6], while Pratt et al. [60] offered a particularly detailed discussion of the priority-setting processes required to enable what they characterised as “*deep inclusion*”.

**Table 5:** The role of publics in research priority-setting

Include publics to identify priorities	∙ Need for public involvement in priority-setting at organisational and national levels [1, 7, 15, 16, 56, 60]
	∙ Descriptions of approaches to involving publics in priority-setting [48, 57–59]
	∙ Implications of different structures and processes for involving publics in priority-setting [6, 60]
Coordinate adherence to priorities	∙ Limited engagement of publics in priority-setting
	◦ Research funding agencies do not routinely include publics in research priority-setting [4, 19]
	∙ Limitations to public involvement in redirecting research priorities
	◦ Funding agencies in responsive mode – to policy or researchers [37]
	◦ Public involvement threatens “*established research structures, procedures and cultures*” [1] and priorities of scientists [47]

Strategies for coordinating adherence to research priorities – ensuring and sustaining the impact of public involvement in research priority-setting – were less commonly discussed. Several scholars argued that, with rare exceptions, priority-setting across the research system had been relatively unaffected by public involvement [4, 7, 19, 37], and pointed to the limited capacity for public involvement strategies and processes to redirect “*value-laden and political*” research priorities [60]. Involvement initiatives were often partial [41], and even well-developed efforts might not prove sufficient to redirect research priorities away from dominant concerns to, for example, “*consumer control*” research [4, 37] or action-oriented and intervention research concerned with social determinants [38, 42, 45].

##### The role of public involvement in setting and monitoring ethical standards for health research

Ethical questions are central to the third operational component of the stewardship function given the ethical, legal, economic and social challenges arising with many scientific advances, as well as the persistent challenges in how the risks and rewards of conducting research or allocating its benefits are to be distributed among and between private and public sector actors [27].

The role of public involvement with respect to ethical standards evoked two argument strands (Table 6). First and most clearly, some papers identified a role for publics as ethical arbiters in the conduct of specific projects or research organisations [38, 41, 44, 61]. Second, some papers conceived of a higher order ethical balancing act – one which was provoked by public involvement but did not clearly identify a role for public involvement in its resolution. These authors conceived of public involvement as a powerful challenge to existing authorities and epistemic assumptions about the practice and purpose of health research – a challenge that necessitated a better balance between traditional and newer approaches and normative assumptions [2, 4, 19, 39].

**Table 6:** The role of publics in ethical standard setting

Publics as ethical arbiters	∙ Ensuring ethical conduct in specific projects as well as through advocating for improved ethical review processes or principles [38]
	∙ Public involvement in ethical review bodies (e.g. research ethics boards, institutional review boards) with influence in research systems, and to support public involvement in research (especially community-based participatory research) [41, 44, 61]
Public involvement as ethical balancing act	∙ Public involvement as challenge to essentialist notions of medical science as a form of knowledge that is, and should be, detached from social practices and norms [4, 19, 39]
	∙ Need to reconceive notions of scientific excellence to include societal impact [19] and achieve “*an optimal participation balance*” between publics and other experts [2]

##### Monitoring and evaluating public involvement in the HRS

The final operational component of the stewardship function relates to monitoring and evaluation. Included papers attended to this activity at two levels, namely at the level of the individual project and at the level of the organisation or jurisdiction. Many of the papers called for, described or recommended approaches to monitoring and evaluating public involvement in health research, focusing primarily on public involvement in research projects [4, 5, 47, 59, 62]. A few policy reports advocated monitoring and evaluation of organisations and systems, with recommendations for organisations to monitor and evaluate the public involvement activity that they fostered or pursued, as well as approaches, such as regular review and data collection, to monitor the uptake of public involvement across sectors or systems [17, 63].

Additionally, a number of scholarly papers offered critical reflections on the challenges of evaluating public involvement (Table 7). A few engaged debates about the extent to which an activity motivated by ethical commitments should be judged for its instrumental effectiveness [4]. Others reflected on the challenges of methodology, measurement and intent, given the complexity of public involvement as an intervention, as well as concerns about the evaluative interpretations, methods and processes that might minimise or misunderstand public involvement’s value or impact [4, 19, 40, 61].

**Table 7:** Monitoring and evaluating public involvement

Monitoring public involvement in research projects	∙ The need for formal evaluation and reporting to show the value of public involvement [4, 47, 62]
	∙ Descriptions of approaches taken to monitoring public involvement by research organisations [5, 59]
	∙ Criteria and indicators to support common understanding of expectations [16, 17]
Monitoring public involvement in organisations and systems	∙ Need for organisations to develop and implement strategies to monitor and evaluate their performance [17]
	∙ Strategy for evaluating public involvement across the United Kingdom health research community, involving monitoring and evaluation by research organisations (funders and producers) of the public involvement they support, as well as review of members’, and the wider research sector’s, progress in fostering public involvement [63]
Critical reflection on monitoring and evaluation	∙ Tensions regarding the appropriateness of evaluating public involvement [4]
	∙ Methodological difficulties in evaluating public involvement [40]; the influence of metrics on the interpretation and shape of public involvement practice [19]
	∙ Challenges for adequate comprehension and valuation of the impacts of public involvement [4, 19, 61]; theorising ‘orders’ of change and the meaning of influence or impact [39]

#### Financing

The second major function of a HRS with relevance to governance for public involvement concerns its financing, both in securing funds to support public involvement and in accountably allocating these funds [27].

##### Secure funds to support public involvement in research

The issue of securing funds to support public involvement in research was not discussed in great detail (Table 8). Some scholarly papers identified specific needs for funds for public involvement, advancing the argument that robust public involvement adds to the cost of the research enterprise [46, 57, 58], but that these added costs may be neither anticipated nor appropriately valued [4, 5, 60]. Furthermore, some authors considered how research funds to support public involvement should be secured and managed. Governments and other research organisations (e.g. universities, NGOs) were seen as having a role in securing or coordinating funding to enable public involvement [38, 46, 60]. Finally, funding policy was identified as a relevant factor given the potential significance of stable as compared to competitive funding and the risk of conflicts of interest where funders of public involvement had an interest in specific research outcomes [55].

**Table 8:** Public involvement in securing research funds

Need for funds	∙ Public involvement adds to the cost of the research enterprise – to provide the information, training and infrastructure that publics and researchers require to enable involvement and sustain partnerships, as well as to permit research projects and programmes of research to be conducted at the pace and in the manner that supports meaningful public involvement [46, 57, 58]
	∙ Added costs may not be anticipated or valued [4, 5], especially to support deep inclusion and equity [60]
Source of funds	∙ Governments should allocate sufficient funds [60]
	∙ Research organisations (universities, public or private funding agencies) should raise funds or collaborate to ensure adequate funds [46]
	∙ NGOs can be sources of funds for research, provide in-kind support or identify other funding sources [38]
Funding policy	∙ Funding structure (e.g. more competitive, less stable) may limit capacity for developing and sustaining partnerships with publics [55]
	∙ Funding conflicts of interest – some ways of funding public involvement in health research at particular risk of conflicts of interest, as when a health service funds research that involves service users [55]

##### Define the role of public involvement in accountably allocating funds

In contradistinction to the issue of securing funds, included papers offered considerable insight on how monies should be allocated to support public involvement in research (Table 9). Additionally, unlike Pang et al. [27], who noted the importance of “[a]*n efficient, transparent, and peer-review-based process … at the core of this function*”, these papers emphasised a role for publics – not peers – in fund allocation. Publics could be involved in funding allocation in three ways, namely (1) through participation in grant review processes, (2) through the criteria and calculus used to assess the adequacy of projects and of the public involvement activity embedded within them, and (3) through the types of financial flows and reimbursement mechanisms that could support public involvement in practice.

**Table 9:** Public involvement in allocating research funds

Participation in review	∙ Descriptions of processes used to involve publics in review of research projects [13, 17], including public observation of or participation in scientific peer-review processes, or separate review or ‘triage’ processes [46, 47]
	∙ Analysis of approaches to including publics in research review, including dedicated consumer review panels [53] or public peer reviewers [57–59]
	∙ Analysis of involvement of publics in research review across research systems [5, 19, 64]
Criteria and calculus to allocate funds	∙ Criteria to assess research projects
	◦ Using consumer-identified values and associated guidelines [46, 47]
	◦ Using criteria relevant to community-based participatory research when under review [46, 51]
	∙ Criteria to assess adequacy of public involvement
	◦ Evidence of relevance of public partners and extant engagement [48]
	◦ Evidence of adequacy of time and funding allocated to public involvement [6, 44]
	∙ Calculus to assess evidence of public involvement
	◦ Varied approaches, e.g. mandatory minimums, weighted criteria or un-weighted criteria [5]
	◦ Concern that prevailing evaluative logics render public involvement a secondary consideration [19]
Funding flows	∙ Mechanisms to support researchers to pursue public involvement
	◦ Involvement as condition of funding [54]
	◦ Encourage students/junior researchers through leaves or fellowships [61, 65]
	∙ Mechanisms to enable publics to be involved
	◦ Publics face financial challenges that impede involvement, especially communities and civil society organisations [38, 41, 42, 51, 66]
	◦ Advance planning by researchers to anticipate funding needs, such as training, and expenses incurred by publics, such as travel costs, child care costs, sitting fees for participation [17, 43, 52, 56]
	◦ Challenges in flowing funds to public partners [17, 44, 45]
	◦ Funding arrangements that support public involvement independently of embedded public involvement activities within specific grants [19]
	◦ Funding arrangements where publics are the ‘institution paid’ with support mechanisms to increase capacity for community partners to be successful in securing funds [46, 65]

Among the papers that discussed allocation processes, many were highly descriptive, identifying different ways in which publics could be involved in research review [13, 17, 46, 47]. Others took a more explicitly evaluative approach, analysing approaches to involving publics within specific organisations or across whole research systems [6, 19, 57–59, 64]. These papers were more critical, pointing to the significance of different types of involvement, at different points in the process, for shaping research agendas [6] and the limited extent to which publics were involved in such processes across research funding agencies generally [19, 64].

A number of papers considered the issue of allocation criteria and calculus, identifying different ways in which public involvement might be relevant to the adjudication of research projects. Several papers were concerned with how projects that involved publics would be assessed and called for attention to consumer-identified values and guidelines [46, 47], or the use of criteria that were specifically relevant to community-based participatory research where such research was under review [46, 51]. Other papers were concerned with how evidence of involvement with publics might be considered in research funding decisions. Several papers identified criteria for judging public involvement, such as evidence of the relevance of the specific partnership and the depth of extant involvement, or the adequacy of the time and financial resources allocated to further public involvement activities [6, 44, 48]. Others discussed the calculus to be used in weighing and balancing public involvement as a criterion relative to other criteria. Papers offered descriptions of the varied ways in which funding agencies specified expectations for how involvement was to be described in applications and standards for how these descriptions were to be factored into decisions [5]. Further, some scholars criticised prevalent evaluative logics, which were seen to minimise the importance of public involvement in the adjudication of research projects [19].

Finally, several papers discussed the allocation of funds in terms of the mechanisms through which funds were disbursed, which could be more or less supportive of public involvement in practice. Some of this literature focused on the funding mechanisms that could encourage researchers to pursue public involvement in research. This included funding requirements, such as where public involvement was a condition of funding [54], or funding opportunities (e.g. leaves, fellowships) that might encourage students or junior researchers to pursue public involvement activities [61, 65]. Most of the literature discussed the limitations of existing funding arrangements for enabling publics to engage in research.

That publics face funding challenges that typically impede involvement was highlighted in many papers [38, 41, 42, 51, 66]. To address these challenges, several policy reports discussed the need for researchers to proactively plan for involvement activities in the development of research budgets [17, 43, 52, 56]. Additionally, challenges included mechanisms that could limit the allocation of funds, specifically rules regarding eligibility for receipt of funds, and administrative practices that could significantly delay payment or reimbursement to community partners [17, 44, 45]. Finally, several scholars took issue with the usual approaches to funding public involvement. This included the tendency to fund public involvement where it was embedded within specific research projects rather than as a standalone activity, and to flow funds through academic rather than public participants. Some scholars argued for direct funding for publics to enable them to partner in health research, including by permitting a community partner to be the grant recipient, and by providing pre-application support to increase the odds of success [46, 65].

### Creating and sustaining resources to conduct, absorb and utilise health research that involves publics

Pang et al. [27] argue that “*creating and sustaining resources remains as a central issue*” for HRS, with a need to produce and sustain human capacity, including through training and the availability of reliable career paths, as well as through physical infrastructure to conduct and use health research. While the creation of capacity clearly requires financial resources, as noted above, we herein review the non-financial dimensions of the resources needed to support public involvement in HRS, distinguishing between human resources on the one hand and organisational arrangements and infrastructure on the other.

Concern with human resources to support public involvement was prominent among included papers (Table 10). A commonly identified strategy to build human resources for public involvement concerned the provision of information or short-term training, for both researchers and publics [4, 41, 46, 47, 52, 57–59, 63, 64, 67]. This was needed to provide basic knowledge about research and partnerships working and necessitated that individuals be provided with access and that research organisations ensure availability [41, 43, 47, 56].

**Table 10:** Human resources for public involvement

Human resource needs	∙ Information and training to equip researchers and publics
	◦ Communication skills, partnership working [52, 63, 67]
	◦ Conduct and organisation of research, specific research tasks (e.g. priority-setting, ethics review) [4, 46, 47, 57–59, 64]
	◦ Availability of resources, content of resources, guidance for research organisations on incorporating training into organisation-wide initiatives [41, 43, 47, 56]
	∙ Sustained support to empower publics to contribute substantively
	◦ Induction and training programmes, mentorship and feedback schemes, with dedicated staff time and space [54, 55, 59, 62, 68]
	◦ Especially important for ‘deep inclusion’ [60]
	∙ Organisational effort to encourage researchers to engage in partnerships with publics
	◦ Education: recruit students from marginalised and ‘studied’ communities [46], teach principles and practice of community-based participatory research [51]
	◦ Mentorship, supportive networks and the need for critical mass [44, 61, 65, 69]
	◦ Recognise and value research involving publics: performance reviews and promotions criteria [44, 46, 61, 63, 65]

Yet, the need for support was not only initial or temporary but included longer-term mentorship or support and necessitated committed effort by research organisations. Ongoing mentorship and feedback was needed to enable publics to participate actively and contribute substantively [54, 55, 59, 62, 68], and was especially important to permit meaningful inclusion by members of more disadvantaged communities [60]. To make such support available, research organisations needed to commit resources, including staff time and space [54, 55, 59, 62, 68]. Finally, there was a need for specific efforts to encourage researchers to involve publics in research. Research capacity could be created through graduate programmes, which could provide training in the principles and practice of public involvement in research and might recruit students from ‘studied’ communities to become future researchers [46, 51]. There was also a need to sustain research capacity through opportunities for mentorship from senior researchers with expertise in public involvement and access to supportive networks and a ‘critical mass’ of committed colleagues [44, 61, 65, 69]. Finally, support for researchers necessitated the appropriate valuation of public involvement in professional advancement metrics and processes, given concerns about the extent to which usual tenure and promotion processes and metrics for ‘impact’ devalued the types of applied, often-local and time-consuming work that meaningful public involvement required [44, 46, 61, 63, 65].

The resources required to ensure that individual researchers and members of the public were informed and empowered to collaborate necessarily implicated organisational practices, as already suggested. However, capacity-building needs were also expressly organisational in nature, including consideration of how commitments to public involvement could be organisationally instantiated and shared across the research system (Table 11). One area of organisational practice that was regularly identified related to the mobilisation of advocates for public involvement, including through roles for managers and other public involvement professionals, or through the organisational arrangements that gave regular voice to publics in research organisations [54, 56, 63, 68]. A second area of organisational practice was related to research infrastructure to enable the conduct of research that involved publics. In part, this related to organisational reform to alter the operation of research-producing organisations through restructured academic committees, as well as support for novel research positions, organisations, networks or nodal capacity to serve as the coordinators, conveners, doers, resource brokers and all-round capacity-builders for public involvement in health research [61, 62, 65, 68].

**Table 11:** Organisational capacity and infrastructure for public involvement

Capacity in organisations	∙ Mobilising advocates of public involvement
	◦ Positioning of supporters of public involvement: senior leaders, managers or professional facilitators as ‘organisational drivers’ [54, 56, 63, 68]
	◦ Positioning of publics: advisory groups and advocate roles and offices [54, 68]
	∙ Supportive research infrastructure within research organisations
	◦ Reform to usual organisational arrangements, such as academic committees and committee memberships (e.g. tenure and promotion committees, ethical review committees) [61, 65]
	◦ Novel organisational forms, e.g. organisations led by service-user researchers, owned and stewarded by communities [68], arrangements for specific communities (e.g. Aboriginal Health Research) [62], creation of joint research appointments (community, research organisations), nodal networks or centres [61]
Capacity for research systems	∙ Mobilising advocates of public involvement
	◦ Strategic and structural positioning of publics in key research organisations and roles: “*structurally involved in formal decision-making processes*” [2] at “*strategic level*” [37] (see also [39, 51])
	∙ Supportive research infrastructure across research systems
	◦ Infrastructure to overcome “*fragmented and uncoordinated*” structures for involvement [55], such as a single access point or ‘portal’ for publics [17], or state-wide registers of consumers, which could aid researchers and offer match-making [68]
	◦ Infrastructure developed or supported by states or major research, e.g. in United Kingdom, the Consumers in NHS Research Support Unit [4], INVOLVE, James Lind Alliance [40], or developed collaboratively for “*economies of effort*” for those providing training or engaging in consultation, and to minimise the challenge of “*consultation fatigue*” [57]
	∙ Challenges of overly professionalised public involvement infrastructure – “*thriving and burgeoning public involvement infrastructure*” with a “*new strata of jobs with titles such as ‘Public Involvement Lead/Facilitator/Adviser/Coordinator’*” that had not supported a shift in “*power to the people*” [37]

Organisational capacity was also needed beyond the walls of individual research-producing organisations to serve the research system as a whole. This involved mobilising advocates of public involvement in structural and strategic ways, with formal positions for publics within key research organisations [2, 37, 39, 51]; it also involved the creation of infrastructure that could be jurisdiction-wide in its relevance and impact. Such infrastructure could help to coordinate the involvement of publics in priority-setting or provide coordinated training and support to publics and public involvement. Furthermore, system-wide capacity was discussed as a means of overcoming fragmentation of effort, for example, through the creation of single access points or ‘matchmaking’ services for publics and researchers [55, 68]. Such infrastructure might be developed by states or major research organisations or emerge through collaboration [4, 40, 57]. Against this, some authors cautioned against the risk that an overly professionalised public involvement infrastructure might side-line publics, and the more radical demands that diverse publics might articulate [37].

### Producing, synthesising and utilising research

In general, attention to research use was limited in the documents we consulted, though the literature is clear in perceiving involvement as a key force in ensuring that the right research, asking the right questions, is done in the right way, so that it can be used. Publics provide expertise, judgment and capacity in knowledge production and are seen as key beneficiaries and users of – as well as advocates for the use of – health research [38, 60]. Indeed, the public commitment to research use may be more developed than is the case for other participants in the research process, including academics, universities or funding organisations.

## Discussion

Increased involvement of publics in health research has been accompanied by increased effort by governments and research organisations to foster, direct and evaluate the public involvement enterprise. This raises critical questions about how and with what effect public involvement has been embedded within organisations and jurisdictions – questions that policy-oriented scholars have begun to pose.

We approached these policy questions from the perspective of governance – asking how the rules, norms and actions that structure and sustain public involvement in health research are coordinated and held accountable within jurisdictions. Central to our effort was an established framework designed to support countries to strengthen their HRS [27]. This framework identified four core ‘functions’ of a HRS, and nine embedded ‘operational components’, which served as the data collection architecture for our search, selection and analysis of scholarly papers and policy reports. Additionally, it provided a centrally important concept – that of the HRS itself – to define the space within which governance effort is embedded.

Though newer and less well known, the concept of the HRS is as important as the complementary concept of the ‘health system’. Both terms call attention to the interconnected sets of organisational and individual actors and the web of rules, expectations and structures that produce health research or health services and population health outcomes, namely research, services or outcomes that are more or less robust, relevant and responsive. Where system theories that emphasise complexity and adaptation are drawn on, these concepts also highlight the dynamic and emergent nature of many system properties and the potential for both intended and unintended effects [23, 70–72], and suggest the value of longitudinal research that queries not only the achievement of the desired visions and intended outcomes of public involvement initiatives, but also the unintended effects of these initiatives on the health and healthcare of actors and the subsystems in which they are embedded.

A governance perspective has considerable value for public involvement in HRS, supporting efforts to coordinate and institutionalise the burgeoning public involvement enterprise. The perspective points, first, to the range of ways in which public involvement has to be enacted by organisations and within jurisdictions to support and give full effect to the efforts of countless individuals. This involves stewardship to articulate a compelling vision for public involvement, to clarify the ways in which publics will set research priorities and ethical standards, and to foster clarity and consistency in the monitoring and evaluation of public involvement. This also involves financing to ensure the adequacy of funds to support public involvement and to address issues in funding policy, including the structure of funding flows and the potential for conflicted expectations by funding sources. This further involves capacity-building, in both human and organisational terms, to empower individual researchers and publics to partner and build commitment to, and enable, public involvement within research organisations and across research systems. Finally, this involves attention to the cycle of research production, synthesis and use – in recognition of the public commitment to research relevance and impact [73], and the role of public involvement in ensuring such outcomes.

The evidence collected and organised here might be used by public involvement leaders and research system stewards to catalyse reflection and action. It is relevant also to public involvement researchers to encourage existing efforts to forge system-wide approaches on critical issues such as evaluation [74, 75], and to identify the need for more policy-oriented work to compare, assess and theorise about the operation and effects of varied organisational and jurisdictional efforts across all HRS functions.

Yet, a governance perspective does not simply identify domains of activity and opportunities for research. It also highlights challenges for what is, ultimately, a highly political intervention. As many scholars have noted, the aim of public involvement is contested – whether to enable to ‘do better’ what is already done, or to challenge existing assumptions and arrangements, including about the nature and epistemic authority of science [37, 39]. Contestation over the ends of public involvement was clearly evident in our review, particularly with respect to the stewardship function. Importantly, visions for public involvement differ with respect to both ‘who’ to involve and ‘how’. Further, differences extend beyond goal-setting to encompass the question of implementation and the ultimate impact of public involvement in health research.

The differences in vision that our review highlighted have important implications for HRS and the health systems they seek to serve. In particular, the scholarly literature is largely split between discussions of communities on the one hand, and patients on the other, while policy reports are primarily concerned with patients. Relatedly, scholarly literature on patient involvement emphasises high levels of involvement and scholarship on community involvement emphasises a specifically participatory and action-oriented approach, while policy reports are typically agnostic, allowing the notion that involvement can extend from research partnership through to research participation. Terminological differences are relevant here; in some contexts, the term ‘involvement’ implies more than ‘engagement’, while in others the reverse is the case [4, 76, 77]. Additionally, the term ‘public’ is not necessarily recognised or welcomed by the individuals to be involved, who may instead identify as patients, clients, service users, family members, community members or activists [78].

Nevertheless, the differences we identified extend beyond terminology to the politics of public involvement itself. Public involvement that aims towards partnership and action is potentially transformative, whereas public involvement that aims at public participation is not. Further, which ‘publics’ are to be involved has implications for what expertise will be mobilised and thus what research will be prioritised and pursued. If patients are only asked to represent themselves as individuals, and not as and with diverse communities nor as and with lay publics, then the aims of HRS to support health equity and to enable health beyond the delivery of care, including through public health efforts and ‘health in all’ policies and practice, will be abridged.

In addition to contestation over visions, our review identified concerns about the particular and partial implementation of the public involvement agenda and its limited impact. Visions for public involvement may be implemented to advance the self-interest of the research organisation rather than foster public empowerment [19], and even the impact of transformative visions may be conditioned by resistant organisational arrangements and network processes [2]. Furthermore, public involvement has seen partial uptake across funding organisations and its impact on redirecting research priorities remains limited [41, 60].

Issues of implementation and impact become highly visible from a health research ‘systems’ perspective but raise profound challenges for governance and accountability. Both in theory and in practice, the governance of research systems is a distributed function that mobilises many authoritative actors. Indeed, as Caron-Flinterman et al. [2] highlighted in an early example of policy scholarship on public involvement, multiple ‘intermediary organisations’, such as research funding agencies, research producer organisations (i.e. universities, hospitals) and public involvement support organisations (e.g. INVOLVE), are positioned between science and society and structure opportunities for public involvement effort and impact. As more recent research on regulation and science policy makes clear, such critical ‘intermediaries’ are not simply passive channels for transferring knowledge and objects between science, policy and economy [79], but rather “*they mobilize, reframe and structure expertise and policy imperatives*” [80].

Governance challenges within networked systems are not unique to public involvement, though they assume a specific form. Given the important role of powerful intermediary organisations in HRS, questions arise about how accountability expectations for public involvement are to be established, how they are to be exercised (specifically what policy instruments or tools can be used) and how and with what transparency and traceability they can be enforced. Scholars who have addressed the challenges of governance and accountability for health systems highlight the need to emphasise the distinctive legitimacy of governments as authoritative actors and the importance of financial instruments in driving coordinated action and outcomes [22]. These remedies may have relevance to the governance of HRS in general and to public involvement for such systems in particular.

The political nature of the public involvement agenda and the governance challenges that arise for HRS highlight an additional dimension of public involvement – one that we did not anticipate. The initial aim of our review was to explore how to govern for public involvement ‘within’ a HRS, which we understood as ensuring that support for public involvement is embedded ‘system wide’ and that ‘systemic effort’ is made to ensure its effectiveness. Yet, our review also highlighted a slightly different but equally important governance question – that is, what is the ‘role’ of public involvement in ‘governing’ HRS. Here, the question is less about how to embed public involvement through policy, process or infrastructure, and more about the importance of public involvement for navigating tensions within, and legitimising the efforts of, a HRS. Contests over vision and issues of accountability in the implementation and impact of public involvement cannot be resolved by researchers and the traditional governors of the research enterprise. Judgments of this sort, as Milewa et al. [39] have argued in a related context, “*can only rest upon discursive processes within the social realm*”.

The limitations of this review are several. Of note, time and resource constraints significantly limited our evidence review. We systematically searched only one database and supplemented our collection through backward and forward citation searches of included papers, and the collection of relevant papers from the investigators’ files. The limitations of our restricted search were likely compounded by the use of MEDLINE, which does not reference all relevant social science literature, the weakness of MeSH terms for identifying public involvement scholarship, and the importance of grey literature for reports on public involvement. Our targeted environmental scan aimed to address some of the grey literature limitations, but it too was limited by the restricted number of jurisdictions and organisations targeted and our focus on specific types of policy reports.

While searches across additional databases would be a partial remedy, we would suggest that a greater limitation may arise from our focus on policy-relevant scholarship, specifically the requirement that papers be focused on research systems, networks, organisations or jurisdictions to be eligible for inclusion. While such a selection criterion was necessary to make our search tractable, it is likely that a much broader literature set would be relevant to the issues of governance that were the focus of our review. This is perhaps especially notable in relation to the fourth function of the HRS – producing/using health research – for which we identified very little research. A much more expansive review or, more feasibly, a series of reviews focused on specific research system functions, would add significantly to the evidence collected and might highlight governance issues not seen through this review.

## Conclusions

Despite limitations, the suggested framework for governance for public involvement in HRS provides both insights and guidance. One conclusion must certainly be the need for more policy-oriented research on public involvement; specifically, research that compares, contrasts and evaluates the varied processes, arrangements and initiatives that mobilise public involvement within and across organisations and research systems. Additionally, we offer insight into the role of publics in HRS, not simply as components of a HRS, but as a central plank in its good governance.

## Additional files

Additional file 1: Search terms. (DOCX 32 kb)

Additional file 2: List of key organisations included in environmental scan by jurisdiction. (DOCX 21 kb)

Additional file 3: List of included journal papers. (DOCX 20 kb)

Additional file 4: List of included policy reports by jurisdiction. (DOCX 16 kb)

Additional file 5: Conceptualising patient and public involvement [1, 4, 6, 15, 16, 38, 41–48, 51, 56, 61–63, 81]. (DOCX 17 kb)

## Abbreviations

HRS: health research systems
MeSH: Medical subject heading
