# Supplementary material for: Public involvement in health research systems: a governance framework
Source: Health Res Policy Syst. 2018 Aug 6;16:79. doi: 10.1186/s12961-018-0352-7 (PMC6080531; doi:10.1186/s12961-018-0352-7)
Supplement: Supplementary file 1 — Search terms. (DOCX 32 kb) [file 12961_2018_352_MOESM1_ESM.docx]

**Medline**

(consumer participation/ or patient participation/ OR ((advocacy or advocat* or participat* or engag*) adj3 (consumer* or patient* or public)).ti,ab,kf. OR Patient-Centered Care/) AND ("Organization and Administration"/ OR capacity building/ OR "Diffusion of Innovation"/ OR Knowledge Management/ OR health services research/ or organizational case studies/)
