# Supplementary material for: Public involvement in health research systems: a governance framework
Source: Health Res Policy Syst. 2018 Aug 6;16:79. doi: 10.1186/s12961-018-0352-7 (PMC6080531; doi:10.1186/s12961-018-0352-7)
Supplement: Supplementary file 2 — List of key organisations included in environmental scan by jurisdiction. (DOCX 21 kb) [file 12961_2018_352_MOESM2_ESM.docx]

Additional file 2 List of key organisations included in environmental scan by jurisdiction

| Funding agencies | Engagement support organisations |
| --- | --- |
| *United Kingdom* | |
| National Institute for Health Research (NIHR) (INVOLVE) <http://www.nihr.ac.uk/about/> | INVOLVE (NIHR) <http://www.invo.org.uk/> |
| - NIHR is funded by the Department of Health to improve the health and wealth of the nation through research and represents the most integrated clinical research system in the world, driving research from bench to bedside for the benefit of patients and the economy | - INVOLVE is a national advisory group created and funded by the NIHR to support involvement in NHS, public health and social care research, with the aim of advancing it as part of the process by which research is identified, prioritised, designed, conducted and disseminated |
| Research Councils UK (RCUK) <http://www.rcuk.ac.uk/> | National Coordinating Centre for Public Engagement (NCCPE) <http://www.publicengagement.ac.uk/> |
| - RCUK supports research that has an impact on the growth, prosperity and wellbeing of the United Kingdom as well as the training and career development of researchers to engage the wider public with research | - NCCPE is funded by the four United Kingdom Funding Councils, Research Councils UK, and the Wellcome Trust and helps inspire and support universities to engage with the public |
| Medical Research Council (MRC) <http://www.mrc.ac.uk/> | James Lind Alliance (JLA) <http://www.lindalliance.org> |
| - MRC is a non-departmental public body, funded through the government’s science and research budget, that funds research across the biomedical spectrum, from fundamental lab-based science to clinical trials and in all major disease areas | - JLA is a non-profit-making initiative that brings patients, carers and clinicians together in priority-setting partnerships to identify and prioritise the top 10 uncertainties about the effects of treatments that they agree are most important |
| *United States* | |
| Patient-Centered Outcomes Research Institute (PCORI) <http://www.pcori.org/> | Consumers United for Evidence-based Healthcare <http://us.cochrane.org/about-cue> |
| - PCORI is a not-for-profit organisation established by Congress in the Patient Protection and Affordable Care Act that funds comparative clinical effectiveness research aimed to improve the quality and relevance of evidence available to help patients, caregivers, clinicians, employers, insurers and policy-makers make informed health decisions |  |
| National Institutes of Health (NIH) <https://www.nih.gov/> |  |
| - NIH is part of the United States Department of Health and Human Services and is the primary governmental agency responsible for biomedical and health-related research, conducting its own scientific research as well as providing major biomedical research funding |  |
| Agency for Healthcare Research and Quality (AHRQ) <http://www.ahrq.gov/> |  |
| - AHRQ operates within the United States Department of Health and Human Services and aims to improve the safety and quality of America’s healthcare system through developing the knowledge, tools and data needed to do so |  |
| *Australia* | |
| National Health and Medical Research Council (NHMRC) <https://www.nhmrc.gov.au/> | Consumers’ Health Forum of Australia (CHF) <https://www.chf.org.au/> |
| - NHMRC is the major governmental body responsible for supporting health and medical research as well as providing health advice to the Australian community, health professionals and governments | - CHF provides a strong national voice for health consumers and supports consumer participation in health policy and programme decision-making |
| *Canada* | |
| Canadian Institutes of Health Research (CIHR) [includes Strategy for Patient-Oriented Research (SPOR)] <http://www.cihr-irsc.gc.ca/e/193.html>; <http://www.cihr-irsc.gc.ca/e/41204.html> | Patients Canada <http://www.patientscanada.ca/> |
| - CIHR is the major federal agency responsible for funding health research in Canada and provides leadership and support to health researchers and trainees to create new scientific knowledge and to enable its translation into improved health, more effective health services and products, as well as a strengthened healthcare system | - Patients Canada is an independent charitable organisation that fosters collaboration between patients, family caregivers and the healthcare community |
| - SPOR is a coalition of federal, provincial and territorial partners – all dedicated to the integration of research into care and is made up of 10 locally accessible SUPPORT UNITS that lead and facilitate decision-making within the health services setting, foster the implementation of best practices and promote collaboration among researchers engaged in patient-oriented research | - Patient and Community Engagement Research (PaCER) <http://www.pacerinnovates.ca/> |
|  | - PaCER is a joint research training programme between Alberta Health Services and the Institute for Public Health at the University of Calgary, which trains patients, former patients, caregivers and/or family members to become patient engagement researchers in designing and conducting health experience research as well as to work in collaboration with health providers, planners and researchers |
