# Supplementary material for: Public involvement in health research systems: a governance framework
Source: Health Res Policy Syst. 2018 Aug 6;16:79. doi: 10.1186/s12961-018-0352-7 (PMC6080531; doi:10.1186/s12961-018-0352-7)
Supplement: Supplementary file 3 — List of included journal papers. (DOCX 20 kb) [file 12961_2018_352_MOESM3_ESM.docx]

Additional file 3 List of included journal papers

| Ahmed, S.M., Beck, B., Maurana, C.A., Newton, G. (2004). Overcoming barriers to effective community-based participatory research in US medical schools. *Education for Health*, *17*(2):141–151. |
| --- |
| Ahmed, S.M. and Palermo, A.G.S. (2010). Community engagement in research: Frameworks for education and peer review. *American Journal of Public Health*, *100*(8): 1380–1387. |
| Beinare, D. and McCarthy, M. (2012). Civil society organizations, social innovation and health research in Europe. *The European Journal of Public Health*, *22*(6): 889–893. |
| Boote, J., Telford, R. and Cooper, C. (2002). Consumer involvement in health research: a review and research agenda. *Health Policy*, 61(2): 213–236. |
| Caron-Flinterman, J.F., Broerse, J.E. and Bunders, J.F. (2005). The experiential knowledge of patients: a new resource for biomedical research? *Social Science & Medicine*, 60(11): 2575–84. |
| Caron-Flinterman, J.F., Broerse, J.E. and Bunders, J.F. (2007). Patient partnership in decision-making on biomedical research changing the network. *Science, Technology & Human Values*, 32(3): 339–68. |
| Cowan, K. (2010). The James Lind Alliance: Tackling treatment uncertainties together. *The Journal of Ambulatory Care Management*, *33*(3): 241–248. |
| Delisle, H., Roberts, J.H., Munro, M., Jones, L. and Gyorkos, T.W. (2005). The role of NGOs in global health research for development. *Health Research Policy and Systems*, *3*(1): 3. |
| Evans, D. (2014). Patient and public involvement in research in the English NHS: a documentary analysis of the complex interplay of evidence and policy. *Evidence and Policy*, 10(3): 361–377. |
| Fleurence, R., Selby, J.V., Odom-Walker, K., Hunt, G., Meltzer, D., Slutsky, J.R. and Yancy, C. (2013). How the Patient-Centered Outcomes Research Institute is engaging patients and others in shaping its research agenda. *Health Affairs*, *32*(2): 393–400. |
| Green, G. (2016). Power to the people: To what extent has public involvement in applied health research achieved this? *Research Involvement and Engagement*, *2*(1): 28. |
| Israel, B.A., Schulz, A.J., Parker, E.A. and Becker, A.B. (2001). Community-based participatory research: Policy recommendations for promoting a partnership approach in health research. *Education for health*, *14*(2):182–197. |
| Knabe, A. and McCarthy, M. (2012). Civil Society Organizations and Public Health Research-Evidence from Eight European Union New Member States. *Central European Journal of Public Health*, *20*(4): 287–293. |
| Lloyd Michener, M., Cook, J., Ahmed, S.M., Yonas, M.A., Coyne-Beasley, T. and Aguilar-Gaxiola, S. (2012). Aligning the goals of community-engaged research: Why and how academic health centers can successfully engage with communities to improve health. *Academic Medicine*, *87*(3): 285–291. |
| Milewa, T., Buxton, M. and Hanney, S. (2008). Lay involvement in the public funding of medical research: Expertise and counter-expertise in empirical and analytical perspective. *Critical Public Health*, 18(3): 357–366. |
| Minogue, V. and Girdlestone, J. (2010). Building capacity for service user and carer involvement in research: The implications and impact of best research for best health. *International Journal of Health Care Quality Assurance*, *23*(4): 422–435. |
| Moran, R. and Davidson, P. (2011). An uneven spread: a review of public involvement in the National Institute of Health Research’s Health Technology Assessment program. *International Journal of Technology Assessment in Health Care*, *27*(4): 343–347. |
| Nyden, P. (2003). Academic Incentives for Faculty Participation in Community-based Participatory Research. *Journal of General Internal Medicine*, *18*(7): 576–585. |
| O’Donnell, M. and Entwistle, V. (2004). Consumer involvement in decisions about what health-related research is funded. *Health Policy*, *70*(3): 281–290. |
| O’Donnell, M. and Entwistle, V. (2004). Consumer involvement in research projects: The activities of research funders. *Health Policy*, *69*(2): 229–238. |
| Oliver, S., Milne, R., Bradburn, J., Buchanan, P., Kerridge, L., Walley, T. and Gabbay, J. (2001). Involving consumers in a needs-led research programme: a pilot project. *Health Expectations*, *4*(1): 18–28. |
| Petit-Zeman, S., Philpots, E. and Denegri, S. (2010). “Natural ground” for medical research charities: Public and patient involvement in research funding. *The Journal of Ambulatory Care Management*, *33*(3): 249–256. |
| Pinto, R.M. (2009). Community perspectives on factors that influence collaboration in public health research. *Health Education &* *Behavior*. Epub Feb 9, 2009; doi:[10.1177/1090198108328328](http://dx.doi.org/10.1177/1090198108328328). |
| Pratt, B., Merritt, M. and Hyder, A.A. (2016) Towards deep inclusion for equity-oriented health research priority-setting: A working model. *Social Science & Medicine*, 151: 215–224. |
| Royle, J. and Oliver, S. (2004). Consumer involvement in the health technology assessment program. *International Journal of Technology Assessment in Health Care*, *20*(4): 493–497. |
| Sanders, D., Labonte, R., Baum, F. and Chopra, M. (2004). Making research matter: A civil society perspective on health research. *Bulletin of the World Health Organization*, *82*(10): 757–763. |
| Saunders, C., Crossing, S., Girgis, A., Butow, P. and Penman, A. (2007). Operationalizing a model framework for consumer and community participation in health and medical research. *Australia and New Zealand Health Policy*, *4*(1): 13. |
| Saunders, C. and Girgis, A. (2010). Status, challenges and facilitators of consumer involvement in Australian health and medical research. *Health Research Policy Systems*, *8*(34): 10–1186. |
| Saunders, C. and Girgis, A. (2011). Enriching health research through consumer involvement - learning through atypical exemplars. *Health Promotion Journal of Australia*, *22*(3): 196–202. |
| Van Bekkum, J.E. and Hilton, S. (2014). UK research funding bodies’ views towards public participation in health-related research decisions: an exploratory study. *BMC Health Services Research*, *14*(1): 318. |
| Van Bekkum, J. E., Fergie, G.M. and Hilton, S. (2016). Health and medical research funding agencies’ promotion of public engagement within research: A qualitative interview study exploring the United Kingdom context. *Health Research Policy and Systems*, *14*(1): 23. |
| Venuta, R. and Graham, I.D. (2010). Involving citizens and patients in health research. *The Journal of Ambulatory Care Management*, *33*(3): 215–222. |
