# Supplementary material for: Public involvement in health research systems: a governance framework
Source: Health Res Policy Syst. 2018 Aug 6;16:79. doi: 10.1186/s12961-018-0352-7 (PMC6080531; doi:10.1186/s12961-018-0352-7)
Supplement: Supplementary file 4 — List of included policy reports by jurisdiction. (DOCX 16 kb) [file 12961_2018_352_MOESM4_ESM.docx]

Additional file 4 List of included policy reports by jurisdiction

| Funding agencies | Engagement support organisations |
| --- | --- |
| *United Kingdom* | |
| National Institute for Health Research (NIHR) (INVOLVE) | INVOLVE (NIHR) |
| - Going the extra mile: a strategic review of public involvement in the National Institute for Health Research (2015) | - Public involvement in research: values and principles framework (2015) |
| Research Councils UK (RCUK) | National Coordinating Centre for Public Engagement (NCCPE) |
| - Concordat for Engaging the Public with Research (2010) | - Concordat for Engaging the Public with Research: Briefing(s) for senior managers, managers of researchers, supporters of researchers and researchers (2014) |
| *United States* | |
| Patient-Centered Outcomes Research Institute (PCORI) | Consumers United for Evidence-based Healthcare (CUE) |
| - Strategic Plan (2013) | - CUE Bylaws Summary and Mission Statement (2010) |
| - National Priorities for Research and Research Agenda (2012) |  |
| Agency for Healthcare Research and Quality (AHRQ) |  |
| - Effective Health Care Program Stakeholder Guide (2014) |  |
| *Australia* | |
|  | National Health and Medical Research Council (NHMRC) & Consumers’ Health Forum of Australia (CHF) |
|  | - Statement on Consumer and Community Participation in Health and Medical Research (2016) |
|  | - A Model Framework for Consumer and Community Participation in Health and Medical Research (2004) |
| *Canada* | |
| - Canadian Institutes of Health Research (CIHR) [includes Strategy for Patient-Oriented Research (SPOR)] |  |
| - Canada’s Strategy for Patient-Oriented Research: Improving health outcomes through evidence-informed care (2011) |  |
| - CIHR’s Framework for Citizen Engagement (2012) |  |
| - Strategy for Patient-Oriented Research Patient Engagement Framework (2014) |  |
