# Supplementary material for: Public involvement in health research systems: a governance framework
Source: Health Res Policy Syst. 2018 Aug 6;16:79. doi: 10.1186/s12961-018-0352-7 (PMC6080531; doi:10.1186/s12961-018-0352-7)
Supplement: Supplementary file 5 — Conceptualising patient and public involvement [1, 4, 6, 15, 16, 38, 41–48, 51, 56, 61–63, 81]. (DOCX 17 kb) [file 12961_2018_352_MOESM5_ESM.docx]

Additional file 5 Conceptualising patient and public involvement

| Who | Identity | Expertise | Interest in health research | Representation |
| --- | --- | --- | --- | --- |
| Patient | Service users, consumers, caregivers [1, 4, 15, 16, 43] | Experience-based knowledge of clinical conditions, treatments or care pathways [1] | Interest in full range of health research | Represented as core public in scholarly papers |
|  | Potential service users [6, 63, 81] |  |  | Represented as core public in policy reports |
|  | Operationalised as individuals or groups (i.e. patient advocacy organisations) |  |  |  |
| Community | Social constituencies – by common history or interest/identity such as gender, race/ethnicity, sexual orientation, illness experience [44] | Collective expertise – derived from historical, cultural, politico-economic conditions | Interest in population health research, e.g. inequities in care, disparities in outcome [51, 61] | Represented as core public in scholarly papers |
|  | Operationalised as organised or emergent groups, including health, religious or educational organisations [44], civil society organisations or NGOs [38, 41] | To represent a public interest, not the ‘consumer’ of market-based relations [45] | Interest in research beyond health sector for social determinants of health [45] | Generally not identified as core public in policy reports [56] |
|  |  |  | Emphasis on applied, inter-disciplinary research [42, 46] |  |
| Other | Citizen/Public | | | |
|  | - Persons with general lay expertise [47] | | | |
|  | - Includes ‘knowledge users’ such as policy-makers, administrators, community leaders, private sector organisation, etc. [62] | | | |
|  | Clinician | | | |
|  | - Key informants regarding service use, alongside patients [47, 48] | | | |
